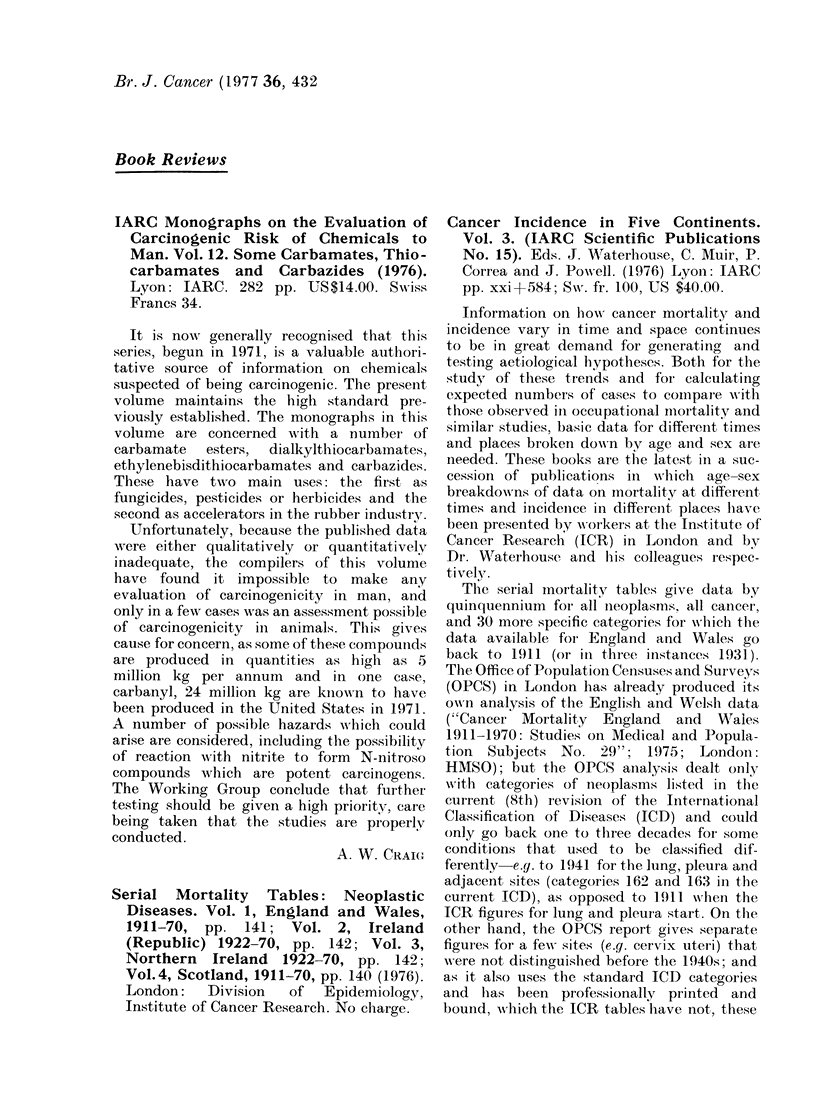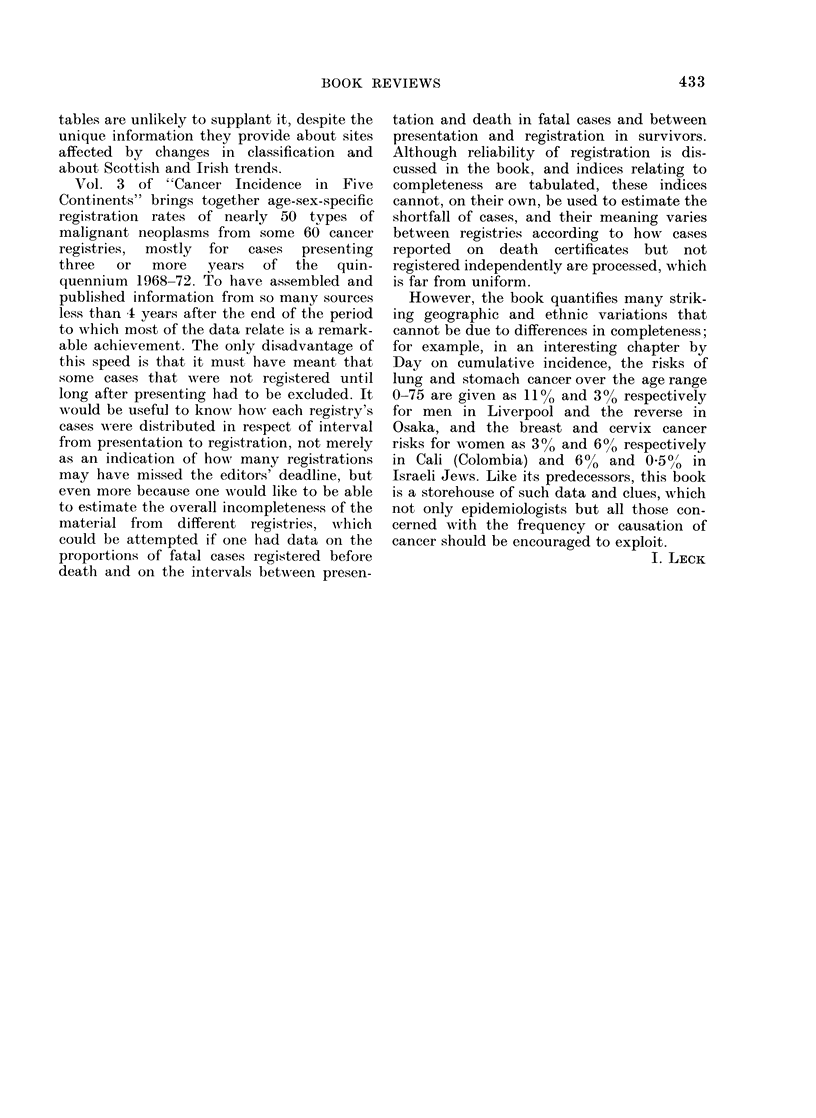# Serial Mortality Tables: Neoplastic Diseases. Vol. 1, England and Wales, 1911-70, pp. 141; Vol. 2, Ireland (Republic) 1922-70, pp. 142; Vol. 3, Northern Ireland 1922-70, pp. 142; Vol. 4, Scotland, 1911-70

**Published:** 1977-09

**Authors:** I. Leck


**Serial Mortality Tables: Neoplastic Diseases. Vol. 1, England and Wales, 1911–70,** pp. 141; **Vol. 2, Ireland (Republic) 1922–70,** pp. 142; **Vol. 3, Northern Ireland 1922–70,** pp. 142; **Vol.4, Scotland, 1911–70,** pp. 140 (1976). London: Division of Epidemiology, Institute of Cancer Research. No charge.

**Cancer Incidence in Five Continents. Vol. 3. (IARC Scientific Publications No. 15).** Eds. J. Waterhouse, C. Muir, P. Correa and J. Powell. (1976) Lyon: IARC pp. xxi+584; Sw. fr. 100, US $40.00.

Information on how cancer mortality and incidence vary in time and space continues to be in great demand for generating and testing aetiological hypotheses. Both for the study of these trends and for calculating expected numbers of cases to compare with those observed in occupational mortality and similar studies, basic data for different times and places broken down by age and sex are needed. These books are the latest in a succession of publications in which age-sex breakdowns of data on mortality at different times and incidence in different places have been presented by workers at the Institute of Cancer Research (ICR) in London and by Dr. Waterhouse and his colleagues respectively.

The serial mortality tables give data by quinquennium for all neoplasms, all cancer, and 30 more specific categories for which the data available for England and Wales go back to 1911 (or in three instances 1931). The Office of Population Censuses and Surveys (OPCS) in London has already produced its own analysis of the English and Welsh data ("Cancer Mortality England and Wales 1911–1970: Studies on Medical and Population Subjects No. 29"; 1975; London: HMSO); but the OPCS analysis dealt only with categories of neoplasms listed in the current (8th) revision of the International Classification of Diseases (ICD) and could only go back one to three decades for some conditions that used to be classified differently—*e.g.* to 1941 for the lung, pleura and adjacent sites (categories 162 and 163 in the current ICD), as opposed to 1911 when the ICR figures for lung and pleura start. On the other hand, the OPCS report gives separate figures for a few sites (*e.g.* cervix uteri) that were not distinguished before the 1940s; and as it also uses the standard ICD categories and has been professionally printed and bound, which the ICR tables have not, these tables are unlikely to supplant it, despite the unique information they provide about sites affected by changes in classification and about Scottish and Irish trends.

Vol. 3 of "Cancer Incidence in Five Continents" brings together age-sex-specific registration rates of nearly 50 types of malignant neoplasms from some 60 cancer registries, mostly for cases presenting three or more years of the quinquennium 1968–72. To have assembled and published information from so many sources less than 4 years after the end of the period to which most of the data relate is a remarkable achievement. The only disadvantage of this speed is that it must have meant that some cases that were not registered until long after presenting had to be excluded. It would be useful to know how each registry's cases were distributed in respect of interval from presentation to registration, not merely as an indication of how many registrations may have missed the editors' deadline, but even more because one would like to be able to estimate the overall incompleteness of the material from different registries, which could be attempted if one had data on the proportions of fatal cases registered before death and on the intervals between presentation and death in fatal cases and between presentation and registration in survivors. Although reliability of registration is discussed in the book, and indices relating to completeness are tabulated, these indices cannot, on their own, be used to estimate the shortfall of cases, and their meaning varies between registries according to how cases reported on death certificates but not registered independently are processed, which is far from uniform.

However, the book quantifies many striking geographic and ethnic variations that cannot be due to differences in completeness; for example, in an interesting chapter by Day on cumulative incidence, the risks of lung and stomach cancer over the age range 0–75 are given as 11% and 3% respectively for men in Liverpool and the reverse in Osaka, and the breast and cervix cancer risks for women as 3% and 6% respectively in Cali (Colombia) and 6% and 0·5% in Israeli Jews. Like its predecessors, this book is a storehouse of such data and clues, which not only epidemiologists but all those concerned with the frequency or causation of cancer should be encouraged to exploit.

I. Leck